# The Cognitive Functions in Adults with Chronic Pain: A Comparative Study

**DOI:** 10.1155/2016/5719380

**Published:** 2016-12-29

**Authors:** Mohammed Shaban Nadar, Zainab Jasem, Fahad S. Manee

**Affiliations:** Occupational Therapy Department, Faculty of Allied Health Sciences, Kuwait University, Jabriya, Kuwait

## Abstract

*Background*. Several studies have reported an association between chronic pain and reduction of cognitive abilities of adults living in Western cultures. No literature could be found on the relationship between chronic pain and cognition among Middle Eastern adults.* Objective*. To compare four of the most commonly reported cognitive domains [memory, attention, processing speed, and executive functioning] among Middle Eastern adults with and without chronic pain.* Methods*. This matched group comparative study included 69 community residing and functionally independent Middle Eastern adults. Forty participants had chronic pain and 29 were pain-free. We administered five standardized cognitive assessments that are independent of culture and language to measure variable tasks of memory, attention, processing speed, and executive functioning. The study was conducted in a rehabilitation research setting with a controlled environment.* Results*. Evidence of decreased cognitive processing was found in patients with chronic pain. The chronic pain participants performed significantly worse than the pain-free participants on the cognitive measures of long-term memory, selective attention, processing speed, and executive functioning.* Conclusion*. The effect of Middle Eastern culture on the cognitive abilities of patients with chronic pain was negligible. Despite the wide variations between Eastern and Western cultures, the performance of our Middle Eastern participants in this study was consistent with performance of Western adults reported in previous studies.

## 1. Introduction

Pain is the unpleasant sensory or emotional experience associated with actual or potential tissue damage or described in terms of such damage [1] and is classified as either acute or chronic. Acute pain is what one feels for a short time after an injury or noxious stimulus and is considered part of a defensive strategy; its specific role is to signal an immediate active danger to the organism [2]. Chronic pain is pain that exceeds the duration of the injury or precipitating stimulus and persists for at least three months [3]. It is surprisingly prevalent among adults: a survey of chronic pain in 16 countries in Europe found 19% of adult Europeans have moderate to severe chronic pain. Very few were managed by pain specialists, and almost half received inadequate pain management [4].

Chronic pain is a complex multidimensional experience that can have a marked effect on many aspects of an individual's daily life beyond physical function [5]. A recent review found that, in addition to the physical sensation itself, chronic pain affects productivity, mood, social life, sleep, participation in leisure activities, and activities of daily living [6]. In addition, it is frequently associated with cognitive impairment [3]. Cognition is a general term for the mental processes that encompasses an individual's ability to process, comprehend, and gain knowledge, including attention, memory, processing speed, judgment, problem-solving, planning, language, imagination, perception, and executive functioning.

Chronic pain itself may not be a direct cause of cognitive impairment, but it may be correlated with the comorbid factors that frequently accompany it, such as emotional distress, anxiety and depressive symptoms [1–5]; care should therefore be taken to avoid direct attribution of pain to accompanying psychogenic factors. A review of clinical and preclinical studies of the effects of pain on cognitive function shows pain to be associated with impaired general cognitive functions [3]. This is most evident in tests assessing executive functioning, attention abilities, processing speed, and memory [4, 5]. These impairments pose further challenges to daily life activities and rehabilitation [3].

The meaning of pain and its toleration vary by culture. Some researchers consider pain as a biopsychocultural experience that affects perception, attitudes to treatment, and expectation of recovery. For example, Irritable Bowel Syndrome is reported more commonly in Western than Eastern women, possibly because of the belief that bowel function is private and can be a source of shame [7]. Somali women are expected not to complain of pain and Somali men are expected to be even more enduring than women are [8]. Similarly, Hispanic women in the USA were found to be more tolerant of pain because their family is given a higher priority over their pain [9]. Islamic cultures in general accept pain and sickness as a source of redemption for past sins [10, 11].

Because most studies that looked at the effects of pain on cognition were conducted in Western cultures, the relationship between chronic pain and cognition could differ in Middle Eastern cultures. Culture is defined as the “patterned behavioral responses that develop over time as a result of imprinting the mind through social and religious structures and intellectual and artistic manifestations” [12, p. 48]. Pain has a cultural meaning; in that culture shapes the beliefs, norms, and the ways people react to and adapt to pain. Evidence that culture has an impact on pain is readily available. In one study, for example, South Asian males showed significantly lower tolerance to thermal pain and experienced a higher intensity of pain than white British males [13]. Similarly, African-American subjects have shown enhanced sensitivity to noxious stimuli and reported higher levels of pain as well as greater pain-related disability than white participants [14]. Libyan students had higher pain pressure thresholds than white British students [15]. Variations in communication style can also influence how pain is expressed and how healthcare professionals respond to patients reporting pain. In a study of 50 hospitalized Arabic patients, the pain ratings were more consistent for those patients cared for by Arabic speaking nurses than when rated by nurses who did not speak Arabic [16]. Situational stress can also influence perceptions of chronic pain. For example, the prevalence of chronic pain in Libya before the Arab Spring uprising in 2011 was 19.6%, similar to the European figure reported above; but it increased to 25% after the uprising [17].

Previous studies in this field have focused on adults with chronic pain living in Western cultures. Of the 29 studies included in the review paper by Moriarty et al. [2011], 16 studies were conducted in Europe, 10 in the USA, and three in Canada [3]. Because no literature could be found on the relationship between chronic pain and cognition among Middle Eastern adults, the purpose of this study was to compare the four most commonly reported cognitive domains [memory, attention, processing speed, and executive functioning] among community dwelling Middle Eastern adults with and without self-reported chronic pain.

## 2. Methods

### 2.1. Study Design

We compared the four selected cognitive domains among community dwelling Middle Eastern adults with and without self-reported chronic pain. Participants were matched by average for the main demographics, including age, gender, marital status, education, and household income. The independent variable was group, and the dependent variables were the cognitive measures: (a) memory, (b) attention, (c) processing speed, and (d) executive functioning. We hypothesized that the chronic pain group would report significantly more pain and have greater cognitive functioning impairments than their pain-free counterparts.

### 2.2. Participants

Sixty-nine Middle Eastern community dwelling adults aged from 18 to 62 years participated in this study. Forty subjects with chronic pain (aged 18 to 62, mean = 39.8 (SD = 12)) made up the “chronic pain” group and 29 pain-free subjects (aged 18 to 54, mean = 34.97 (SD = 10.5)) made up the “pain-free” control group. Chronic pain was defined as daily or almost daily pain of at least moderate intensity (4+ points on the visual analogue scale (VAS) [18]) for more than one year. Pain-free was defined as no pain, rated as zero on the VAS, in the previous week. Participants were excluded if they had visual impairment or a health condition that might affect cognition (such as a psychological or central nervous system problem) or if they were taking opioid medications, which are known to have detrimental effects on cognitive functioning.

### 2.3. Instruments

The independent measure of pain intensity was assessed with a 10 cm visual analogue scale (VAS) by asking the participant, “Please rate your pain by marking the one number that best describes your pain at its WORST in the past week.” Levels of depression, anxiety, stress, and quality of life were similarly measured on a self-reported 10 cm visual analogue scale (VAS), by replacing the word “pain” with depression, anxiety, stress, or quality of life accordingly.

A battery of standardized, empirically validated, and reliable cognitive measures with established psychometric properties was administered to all participants in a fixed order. The order of testing for the cognition measures, the constructs measured, and the methods of administration are shown in Table 1. The cognitive measures were selected because they are independent of culture and language. The measures consisted of five tests (with some containing subtests) that covered the four cognitive domains of memory, attention, processing speed, and executive functioning. The specific outcome measures used (in order of administration in the study) are as follows.

The Contextual Memory Test [19] objectively measures short-term and long-term memory by using pictures of related objects as the items to be remembered. The test consists of picture cards of 20 objects that are related to a specific theme. The participant is shown the cards for 90 seconds and then asked to name the objects seen (short-term memory). Fifteen minutes later, the participant is asked again to name what objects were seen (long-term memory). The Contextual Memory Test is standardized and culturally suitable assessment that is validated for adult 18 years or older [20].

The A Quick Test [21] contains 40 geometric figures [circles, squares, rectangles, or triangles] that are colored in red, black, yellow, or blue. The participant is instructed to quickly name first the color and then the form, in that order, of each stimulus. Color and form must be named in order with the color named first and the shape last (e.g., blue circle and red triangle). The test takes about 5 minutes to administer. It is independent of culture and is a well-validated, sensitive screening tool for cognitive impairment [22].

The Trail Making Test is one of the most widely used instruments in neuropsychological assessment and is included in most test batteries [23]. The test consists of 25 encircled numbers and letters distributed on a sheet of paper. The participant is required to draw lines to connect the circles while sequentially alternating between numbers and letters (like 1, A, 2, B, 3, C, etc.).

The Digit Forward and Backward are among the oldest and most widely used neuropsychological tests of short-term memory capacity [24]. In the Digit Forward Test, the participant is presented with a series of digits (e.g., “6, 3, 5”) and must immediately repeat them back in the same order of presentation. If successful, the participant is given a longer list (e.g., “8, 1, 4, 6”). The length of the longest list a participant can remember is that participant's digit span. In the backward digit-span task, the participant repeats the numbers in a reverse order.

The D2 test of selective and sustained attention [25] includes 14 lines of 47 arbitrarily assorted letters (“p” or “d”) per line. There are one to four dashes over and under each letter. The participant only chooses the letter “d” with two dashes above or below the letter. The participant is allowed only 20 seconds per line before moving to the next line. The D2 test is valid and reliable in measuring attention [26]

### 2.4. Procedure

Ethical approval was obtained from the institutional committee for the protection of human subjects in research, and all participants provided informed consent before beginning the study. Participants completed a demographic form, answered questions about pain (type, cause, and duration), and rated their severity of pain. All cognition measures were administered using standardized directions, and participants were tested in a room with a controlled environment (i.e., sound attenuated, appropriate illumination, and temperature). The total time for completing the cognition measures was about 60 minutes.

### 2.5. Data Analysis

We used descriptive statistics to report the demographic variables and a 1-way ANOVA to test for differences between the demographic variables among groups. Independent *t*-tests were used to compare pain and cognition measures between the two groups, and a Bonferroni correction was used for the clusters of memory variables analyzed together. For heterogeneous variances, we reported the *t*-test results for “equal variances not assumed.” With the Bonferroni corrections, a significant *p* value for the four memory measures had to be *p* < 0.0125 (0.05/4 = 0.0125); the alpha for pain, attention, processing speed, and executive functioning was set at *p* < 0.05.

## 3. Results

The mean pain intensity for the chronic pain group, as measured by the pain visual analogue scale, was 6.35 (SD = 1.6). Chronic pain and pain-free participants were not significantly different with respect to age, gender, marital status, educational level, income, occupation, or psychosocial variables (with the exception of stress) (Table 2). As hypothesized, significant differences between groups were evident in all cognitive domains. The chronic pain group demonstrated greater impairment in long-term memory (*p* = 0.012), selective attention (*p* = 0.002), processing speed (*p* = 0.003), and executive functioning (*p* = 0.029) (Table 3). To examine the influence of the duration of pain experienced by the participants on their cognitive ability, the chronic pain group was divided into two subgroups based on the duration of their pain experience. The first group had less than five years of pain and the second group had more than five years. The groups were compared based on their cognitive function in all domains using independent *t*-test. The results showed no statistically significant differences between these two groups in all cognitive domains (*p* > 0.05).

## 4. Discussion

We utilized five standardized neuropsychological tests that are independent of language and culture to determine the performance of Middle Eastern adults living with chronic pain on four cognitive domains, compared with matched pain-free controls. The results indicate that the participants with chronic pain performed significantly worse in subtests of all four domains: long-term memory, selective attention, processing speed, and executive functioning.

Memory performance is an important aspect of cognition that has been shown to be affected in patients with chronic pain, yet a comprehensive review of 36 studies concludes that not every measure of memory is impaired across all studies. Although chronic pain has been found to affect short-term and working memory in few studies, the most affected memory processes were those involved with long-term memory [27]. In the current study, only long-term memory was impaired in comparison to the pain-free control group. Our results are partly in line with the literature, which found chronic pain to reduce long-term memory, but not short-term and working memory as established in the literature [27–31]. This may be attributed to a number of variables, such as the inconsistency in outcome measures used in the literature, the responsiveness of the measures, or the variability of demand incurred by the task load. Other studies indicate that a decline in memory may be related to the increased depression found in pain patients compared to pain-free controls [29, 32, 33]. Our study found no significant differences in depression scores between those with chronic pain and those who were pain-free.

Of the alternating and selective attention tests conducted in this study, our participants with chronic pain performed significantly worse than the healthy controls in selective attention only. Several investigators have theorized that processing pain requires conscious central attentional control and so subjects with low pain may be able to divert attention from pain to the task at hand, achieving a degree of psychoanalgesia [34, 35]. Pain is an attention-demanding perceptual stimulus, while attention is a limited and unitary resource [34]. It has been suggested that pain competes for the finite attentional resources and thereby affects the performance of tasks that involve the processing and integrating of other information [29, 34, 35]. Ongoing chronic pain may therefore be more likely to disrupt the performance of more demanding tasks, such as in the D2 test, because of its greater aggregate drain on attentional resources. This may explain why our participants performed poorly on the cognitively demanding D2 test, which requires attention and processing speed [25], while did better on the less demanding Trail Making Test of alternating attention.

Previous experimental studies of patients with chronic pain have found attentional deficits associated with high pain severity [34–36]. A comprehensive review of 13 publications on the effects of chronic pain on neuropsychological function found that patients with chronic pain had impaired function in nine of the studies [69.2%], particularly in tests of attentional capacity and processing speed. Similarly, our participants with chronic pain performed significantly worse than the controls in their processing speed, displaying slower reaction times in the D2 measure than their matched controls. This is in line with previous studies that show that patients with chronic pain often show deficits in the speed of information processing [37–39].

The participants with chronic pain in our study also showed impairments in executive functions. Executive functions represent a higher, more abstract level of processing, mainly supported by the prefrontal cortex [40]. A neuroanatomical study observed a loss of cortical grey matter in patients living with chronic pain, especially in the frontal cortices and thalamus [41]. In subsequent studies, a link has been suggested between decreased grey matter in the prefrontal lobe and reduced performance in emotional decision-making [42, 43].

With the exception of stress, the groups in this study showed no significant differences in psychosocial variables, providing support for the notion that deficits in cognitive performance are directly associated with chronic pain. Previous studies have shown that depression, stress, anxiety, and use of opioids are all factors associated with decreased cognitive abilities [36]. Although we controlled for most of these variables, the participants with chronic pain reported greater stress than their pain-free controls, and this could have contributed to the cognitive differences observed between the groups. We therefore cannot rule out the role of stress on cognition in the current study. Nonetheless, brain-imaging studies provide rich evidence of an association between pain and brain areas directly involved in cognitive tasks [44]. Chronic pain is associated with abnormalities in regional cerebral blood flow [45] and with regions involved in emotional decision-making [42]. Collectively, such evidence helps explain how pain impairs cognitive tasks.

Pain is an individualized experience that it is influenced by physiological, social, cultural, and spiritual factors. Culture appears to be a significant factor that influences pain and illness beliefs, behaviors, healthcare practices, help-seeking activities, and receptivity to medical care interventions [46, 47]. Each cultural or religious group has its own unique explanations for the meanings of pain. Although this study does not address cultural perspectives on pain, it is necessary to pinpoint some Middle Eastern Islamic expectations and acceptance of pain as a normal part of life. In Islam, pain is considered to have a positive influence on the soul's prospects in the afterlife; it can be interpreted as a form of divine predestination, and individuals who patiently endure it will have their sins forgiven in the hereafter [10, 11]. Typical behavior of Middle Eastern Muslims may involve reading or listening to the Holy Quran, praying and attending mass, and other involvement with religious groups, to relieve or endure pain [48]. High-quality pain care requires that healthcare professionals view each patient as an individual with many characteristics, including cultural background. Further studies of ethnic and religious conceptions of pain may provide a better understanding of the influence of culture on pain.

### 4.1. Limitations of the Study

It remains difficult to ascribe observed cognitive deficits to a single underlying cause. Our research did not explore in a comprehensive manner the interrelationships among different variables such as pain location, pain duration, sleep deprivation, fatigue, and emotional state; it is thus unclear to what extent these different factors mediate the influence of pain on neuropsychological performance or uniquely contribute to subjective complaints or objective signs of impairment in chronic pain populations. Future studies that include a more comprehensive list of variables will further contribute to knowledge about the impact of chronic pain on cognition.

## 5. Conclusion

The distinctiveness of this study is that it is the first to compare the cognitive performance of Middle Eastern adults with chronic pain to a matched pain-free sample. Previous studies in this field have focused on adults with chronic pain living in Western cultures.

People with chronic pain in the Middle East may be overlooked because cultural attitudes do not consider pain as a medical problem to the same degree that Western cultures do. The deficits perceived in the cognitive performance of Middle Eastern adults with chronic pain in this present study are similar to the accumulated findings of previous studies in Western culture. The similarity of these findings, along with the control for psychosocial variables, suggests that chronic pain contributes directly to cognitive impairments regardless of cultural differences. Cognitive rehabilitation services in the Middle East should broaden their emphasis on patient populations with chronic pain. Failure to assess cognitive function in patients with chronic pain will diminish the likelihood of treating these cognitive shortcomings. Part of the assessment should also include a component on cultural beliefs about pain.

## Figures and Tables

**Table 1:** Description of the cognitive outcome measures (in order of use in the study).

Order	Assessment	Construct[s] measured	Description and method of administration
1	Contextual Memory Test	Short-term memory	The test screens and monitors memory by presenting 20 pictures of related objects for about 90 seconds. The participant is asked to recall the items immediately [short-term memory] and then again after 20 minutes [long-term memory]
2	A Quick Test	Executive functioning	The participant is presented with 40 different stimuli and is asked to name the color [not shape] of each stimulus as fast and as accurately as possible. The scores are recorded in terms of accuracy and time taken for each item set
3	Trail Making Test	Alternating attention	The test requires the participant to draw lines connecting numbers and letters in an alternating and ascending order
4	Digit Forward Test	Short-term memory	The test requires the participant to repeat the numbers heard by the examiner in the same order as given, with number of digits to be remembered increasing with each trial
5	Digit Backward Test	Working memory	The test requires the participant to repeat the numbers heard by the examiner in a reversed (backward) order, with the number of digits increasing with each trial
6	Contextual Memory Test	Long-term memory	See number 1. The participant is asked to recall the items presented 20 minutes ago
7	D2 test	Selective attention Processing Speed	The test requires the examinee to cross out the letters “d” with two dashes, on a sheet with 14 lines, each with 47 characters, for a total of 658 items. Selective attention is the number of errors, and “remaining items” not crossed off are the processing speed

**Table 2:** Demographic and individual variables of the participants.

Demographic variables of participants	Group 1 [chronic pain] *n* = 40	Group 2 [pain-free] *n* = 29	*p* value [cumulative]
Age [years]			
Mean	39.9	35.0	0.086
SD	12.1	10.6	
Gender [*n*, %]			
Male	7 [17.5]	7 [24.0]	0.506
Female	33 [82.5]	22 [76.0]	
Marital status [*n*, %]			
Singled	9 [22.5]	8 [27.6]	0.348
Married	28 [70.0]	19 [65.5]	
Divorced/widow	3 [7.5]	2 [6.8]	
Years of education [*n*, %]			
High school	8 [20.0]	7 [24.1]	0.900
Some college	13 [32.5]	7 [24.1]	
Bachelor or above	19 [47.5]	15 [51.8]	
Income [*n*, %]			
Low	19 [47.5]	12 [41.4]	0.691
Moderate	17 [42.5]	14 [48.3]	
High	4 [10.0]	3 [10.3]	
Occupation [*n*, %]			
House wife	5 [12.5]	2 [6.9]	0.053
Retired [medical]	5 [12.5]	0 [0.0]	
Student	3 [7.5]	5 [17.2]	
Teacher	5 [12.5]	4 [13.8]	
Office work	17 [42.5]	16 [55.2]	
Technician	5 [12.5]	2 [6.9]	
Psychosocial variables [Mean ± SD]			
Depression	4.05 ± 3.2	3.38 ± 2.6	0.365
Anxiety	4.88 ± 2.8	3.59 ± 2.4	0.051
Stress	5.0 ± 3.2	3.4 ± 2.4	0.032^*∗*^
Quality of life	7.28 ± 2.2	7.5 ± 1.7	0.627
Duration of pain [*n*, %]			
Less than 5 years	22 [55.0]	—	—
More than 5 years	18 [45.0]		
Type of pain [*n*, %]			
Musculoskeletal [one joint]	4 [10.0]	—	—
Musculoskeletal [multiple joints]	13 [32.5]		
Musculoskeletal [back only]	8 [20.0]		
Internal [visceral]	5 [12.5]		
Headache	6 [15.0]		
More than one type	4 [10.0]		
Pain medication, nonopioid [*n*, %]			
Yes	18 [45.0]	—	—
No	22 [55.0]		

Note: SD: standard deviation; ^*∗*^*p* < 0.05.

**Table 3:** Results of pain and cognitive measures.

Assessment	Construct measured	Mean ± SD		Sig.	Effect size (Cohen's *d*)
		Chronic pain	Pain-free		
Contextual Memory Test^++^	Short-term memory	14.0 ± 1.7	14.1 ± 3.3	0.821	−.04
A Quick Test^−−^	Executive functioning	5.2 ± 0.9	4.6 ± .7	0.029^*∗*^	.74
Trail Making Test^−−^	Alternating attention	8.3 ± 5.4	6.4 ± 2.2	0.061	.46
Digit Forward Test^++^	Short-term memory	6.9 ± 1.5	7.4 ± 2.1	0.278	−.27
Digit Backward Test^++^	Working memory	4.8 ± 1.6	4.1 ± 1.5	0.065	.45
Contextual Memory Test^++^	Long-term memory	12.4 ± 2.6	14.0 ± 2.3	0.012^*∗*^	.65
D2 test^−−^	Selective attention	15.1 ± 2.6	11.8 ± 4.8	<0.002^*∗*^	.85
	Processing speed	99.7 ± 40.4	71.5 ± 44.2	0.003^*∗*^	.67

^*∗*^Significant *p* value.

^++^Higher score indicates greater performance (less impairment).

^−−^Lower score indicates greater performance (less impairment).

## Additional Points

*Summary*. Several studies have reported an association between chronic pain and the reduction in cognitive abilities of Western adults. This study compares four cognitive domains among Middle Eastern adults: 40 with chronic pain and 29 without, by administering cognitive assessments that are independent of culture and language. Evidence of decreased cognitive processing was found in patients with chronic pain, who performed significantly lower than the pain-free participants. The effect of culture on cognitive abilities seemed minimal, and the performance of our Middle Eastern participants was consistent with the performance of Western adults in previous studies.
